# A phenotype of increased sleepiness in a mouse model of pulmonary hypertension and right ventricular hypertrophy

**DOI:** 10.1371/journal.pone.0208540

**Published:** 2018-12-07

**Authors:** Eric M. Davis, Jeffrey J. Baust, Brett J. O’Donnell, Faraaz A. Shah, Angela McDowell, Lanping Guo, Christopher P. O’Donnell

**Affiliations:** 1 Division of Pulmonary, Allergy and Critical Care Medicine, Department of Medicine, University of Pittsburgh School of Medicine, Pittsburgh, PA, United States of America; 2 Division of Pulmonary and Critical Care, Department of Medicine, University of Virginia, Charlottesville, VA, United States of America; Universita degli Studi di Bologna, ITALY

## Abstract

The relationship between cardiovascular disease and abnormalities in sleep architecture is complex and bi-directional. Sleep disordered breathing (SDB) often confounds human studies examining sleep in the setting of heart failure, and the independent impact of isolated right or left heart failure on sleep is difficult to assess. We utilized an animal model of right heart failure using pulmonary artery banding (PAB) in mice to examine the causal effect of right heart failure on sleep architecture. Four weeks after PAB or sham (control) surgery, sleep was measured by polysomnography for 48 hours and right ventricular (RV) hypertrophy confirmed prior to sacrifice. PAB resulted in right ventricular hypertrophy based on a 30% increase in the Fulton Index (p < 0.01). After PAB, mice spent significantly more time in NREM sleep compared to the control group over a 24 hour period (53.5 ± 1.5% vs. 46.6 ± 1.4%; p < 0.01) and exhibited an inability to both cycle into REM sleep and decrease delta density across the light/sleep period. Our results support a phenotype of impaired sleep cycling and increased ‘sleepiness’ in a mouse model of RV dysfunction.

## Introduction

The relationship between cardiovascular disease and abnormalities in sleep architecture is complex and bi-directional. Although it is well-described that insufficient sleep can cause weight gain [1], induce a pro-inflammatory state [2, 3], and create metabolic disturbances [4–6], the impact of abnormal sleep on cardiovascular disease is not well-defined. Patients with heart failure tend to have a greater occurrence of cardiovascular events in the early morning [7–9] and the presence of heart failure is associated with greater degrees of sleep disordered breathing (SDB) [10, 11] and consequent sleep disruption. Moreover, even in the absence of significant SDB, heart failure *per se* has been shown to interfere with the timing and staging of sleep architecture [12]. The presence of comorbidities in the clinical setting of heart failure further confounds our understanding of the complex interactions between cardiac disease and sleep.

We do know that sleep-wake pattern, even in the absence of confounding SDB, can be adversely impacted in the setting of congestive heart failure although the results are mixed [12–14]. Patients with left ventricular (LV) systolic heart failure (ejection fraction of < 45%) exhibit less subjective sleepiness despite reduced total sleep time compared to a control population, independent of SDB severity [15]. Polysomnography results demonstrated significantly greater sleep onset latency, less overall sleep time, and a reduced sleep efficiency in the LV heart failure group [15]. In contrast, there are reports consistent with a “sleepy” phenotype of heart failure characterized by increased sleep time and reduced daytime activity as described by Hastings et al with use of actigraphy [14]. 24 hour actigraphy monitoring on patients with mild to moderate congestive heart failure, with and without SDB, demonstrated that the group with SDB had increased time in bed, poorer sleep quality, and objective findings consistent with a sleepier phenotype despite subjective complaints. More recently, the discordance between objective and subjective parameters of sleepiness in patients with systolic heart failure were reported by Mehra and colleagues [13]. Thus, in the complex clinical setting of LV heart failure there is no definitive sleep phenotype.

Even less is known about the impact of right ventricular (RV) dysfunction on sleep. Given that LV failure can adversely affect RV function, any effects of RV dysfunction *per se* on sleep are likely less confounded than in the setting of LV failure. Thus, as a first step in developing an animal model to examine the relationship between right heart dysfunction and sleep we utilized pulmonary artery banding (PAB) in mice to study the independent effect of RV failure on sleep architecture in the absence of the effects of obesity, SDB, and other co-morbidities of clinical heart failure. PAB is an experimental intervention [16, 17] to increase pulmonary artery pressure, induce right heart remodeling, and simulate the development of chronic RV dysfunction in rodents. Use of the PAB model, in combination with a well-validated polysomnography technique in mice, allowed us to test the hypothesis that isolated pulmonary artery hypertension and RV dysfunction significantly impairs sleep architecture compared to sham-operated control mice. Such differences in sleep architecture may have clinical implications in terms of the impact on quality of life and the development of comorbid medical conditions in patients with RV failure.

## Methods

### Animals

Experiments were performed in adult male C57BL/6J mice at ~13 weeks of age (12.6 ± 0.3 vs. 12.7 ± 0.6 weeks, p = 0.93; n = 7 mice in the control group and n = 10 mice in the pulmonary artery banding (PAB) group). Animal handling and experimentation was conducted ethically and in accordance with approved Institutional Animal Care and Use Committee (IACUC) protocols at the University of Pittsburgh. The University of Pittsburgh IACUC specifically approved this study (Animal Welfare Assurance number A3187-01).

### Housing

Mice were maintained on a 12:12 hour light-dark cycle and were housed in a customized pyramidal cage [7″ (W) x 9″ (H) x 7″ (L)] with continuous access to food and water. The cage was contained inside a light-controlled and sound-dampening chamber [22″ (L) x 16.5″ (H) x 14″ (W)] (BRS/LVE, Laurel, MD). Mice were housed in the same chambers throughout the entire adaptation and experimental period to control for environmental exposure. The polysomnographic tether exited through a 1” diameter hole in the top of the chamber and connected to low friction swivel and an amplifier.

### Protocol and timeline

Mice underwent open thoracotomy at 13 weeks of age for placement of a PAB or sham procedure. Following a 3 week recovery period, the mice underwent surgical implantation of polysomnographic electrodes on day 21, were habituated to the housing environment for one week, and then were tethered to the recording system for 24 hours. Subsequently, EEG and EMG signals were recorded for two consecutive 24-hour periods in each mouse. One week following completion of sleep data recording, each mouse underwent hemodynamic assessment prior to sacrifice.

### Surgical instrumentation

Animals were anesthetized using 1–2% isoflurane for all surgical procedures. Animals, were administered pain medication after surgery (0.3 mg/ml Buprenorphine), and monitored daily during the post-operative period. For PAB, the mice were anesthetized, intubated, and placed on a ventilator (Type845; Harvard Apparatus, Holliston, MA) using a tidal volume of ∼225 μl and a respiratory rate of ∼200 breaths/min. Mice were then placed supine on a heated pad to maintain a body temperature of 37°-39°C (monitored via rectal probe thermometer; THM100; Indus Instruments, Houston, TX). A left thoracotomy was performed and the pulmonary artery (PA) carefully dissected free from the aorta. A surgical clip (Weck 005200; Research Triangle Park, North Carolina, NC) that had been calibrated to a 27-gauge diameter was placed around the PA to create pressure afterload on the RV, as described previously [18]. After banding, the thoracic cavity was closed in layers, and the mice were placed on a heating pad until they regained their righting reflex and became ambulatory. The banding diameter was designed to produce a pressure overload of the RV with an RV end-systolic pressure of ∼30–35 mmHg as per the protocol described by Frazziano et al [18]. The chest was closed and sutured, and the animal was extubated and observed continuously for 2 hours post-procedure and daily afterwards for the first week. Sham control mice underwent the same procedure without placement of the surgical clip around the pulmonary artery.

### EEG and sleep monitoring

Electroencephalographic electrodes (EEG; E363/1, Plastics One, Roanoke, VA) and nuchal electromyographic electrodes (EMG; E363/76, Plastics One) were implanted as previously described [19, 20]. In brief, a midline incision was made to expose the skull and muscles and the underlying fascia was gently cleared from the skull surface, four small burr holes were drilled through the skull in the frontal and parietal regions, and four EEG electrodes were fastened via jewel screws (diameter of 1.6 mm). The first and second electrodes were placed 2–3 mm caudal to bregma and 1–2 mm lateral of the midsagittal suture. The third and fourth electrodes were placed 2–3 mm rostral to bregma and 1–2 mm lateral of the midsagittal suture. Two nuchal EMG electrodes were stitched flat onto the surface of the neck muscle. The EEG and EMG electrodes were inserted into a pedestal (MS363, Plastics One) and secured to the skull with dental acrylic. After recovery from surgery, a connector cable was placed onto the head pedestal and attached to a low-friction mercury swivel allowing unrestricted movement of the tethered mouse.

### Acquisition of hemodynamic data

Approximately one week after completion of sleep data recording, terminal invasive hemodynamic measurements were performed to generate pressure–volume loops, which were used to determine the RV systolic pressure. Induction of anesthesia was achieved in an induction chamber filled using vaporized 3% isoflurane gas. Sufficient level of anesthesia was verified by lack of pedal and corneal reflex and/or lack of response to tail pinch. Animals were ventilated via tracheostomy using positive pressure ventilation after induction of anesthesia with 3% isoflurane gas. A servo-controlled heating pad with rectal probe was used to maintain 37^o^ body temperature. The animals were positioned and secured to the surgical table and surface ECG electrodes placed under each limb using subcutaneous 26G needle electrodes. Longitudinal dissection of the sternum exposed the heart and great blood vessels. The RV was cannulated with a calibrated 1.5F micro pressure-volume catheter (Transonic Systems Inc., Ithaca, NY). The pressure tracing was examined after cannulation while the catheter was gently advanced until the right ventricle systolic pressure (RVSP) was obtained. After data collection was complete, animals were euthanized via terminal bleeding under isoflurane-anesthesia and the RV carefully separated from the LV and septum to calculate the Fulton Index (RV wet weight/LV + septum wet weight) as described previously [18].

### Data acquisition

A Grass Instruments amplifier (Quincy, MA) was used to record EEG activity (filtered 0.1–30 Hz) and EMG activity (filtered 10–100 Hz). Signals from the Grass recorder were collected using Windaq Pro acquisition software (Dataq Instruments; Akron, OH), were digitized at 300 Hz (DI-720 data acquisition board; Dataq Instruments; Akron, OH) and were stored for subsequent analyses.

### Sleep scoring

Sleep data were analyzed using a customized program that converted DATAQ digitized data files into Stanford Sleep Structure Scoring System (SSSSS) format for characterization of signals using the rodent software developed by Joel H. Benington [21], subsequently validated in mice by Veasey et al. [22], and described previously by McDowell et al. [20]. The program utilizes Fourier spectral analysis of the EEG in the delta (0.5–4.0 Hz), sigma (10.0–14.0 Hz), and theta (6.0–9.0 Hz) frequency bands in combination with the moving average of the EMG amplitude to assess sleep/wake states in 10 sec epochs. Twenty-four hour periods of data were plotted as sigma*theta power against EMG, and thresholds for the slope and intercept of the relationship were used to distinguish between sleep and wake. A second plot of the delta/theta power against EMG was used to distinguish non-rapid-eye movement (NREM) sleep from rapid eye movement (REM) sleep on the basis of a delta/theta threshold.

Total time spent in each of the three sleep states (Awake, NREM and REM) was calculated as a percentage of the number of epochs of that state over a 24hr period. The number and duration of NREM sleep bouts and REM sleep bouts were determined according to the following criteria: a NREM sleep bout began with three or more consecutive epochs of NREM sleep and ended with three or more consecutive epochs of wake or two or more consecutive epochs of REM sleep; a REM sleep bout started with two or more consecutive epochs of REM sleep and ended with three or more consecutive epochs of wake or NREM sleep. Time to resume sleep was defined as the length of time from the onset of three or more consecutive epochs of wake to the next combination of any three consecutive epochs of NREM or REM sleep. An arousal was defined as one or more epochs of wake following three or more consecutive periods of either REM or NREM sleep. Arousal frequency per hour of sleep was calculated as total number of arousals / total sleep time in 24hrs. Delta density, marker for sleep pressure, is a term which describes the average delta density during NREM sleep recorded over a set timeframe throughout the sleep recording. It was calculated as power in the 0.5–4.0 Hz range of the Fourier spectral analysis of the EEG during NREM sleep. Delta density was obtained in 2-hour increments within each mouse then reported based on a fraction of the time-point delta density over the average delta density for each mouse for a 24-hour period.

### Statistics

All results are presented as means ± standard error of the mean (SEM) and statistical differences determined with a threshold for significance of > 0.05. Statistical differences between experimental groups for hemodynamic parameters and 12-hr light phase and dark phase sleep data were determined by unpaired Student t-test. Two-way repeated measures ANOVA was used to test for an interaction between intervention and time in different measures of sleep (Awake, REM sleep, NREM sleep, and Delta Density) in the light period and the dark period. Mauchly’s test was applied to test for the assumption of sphericity, and a Geisser-Greenhouse correction was applied if the sphericity assumption was violated. Simple main effects were determined by Fischer’s least significant difference test when repeated measures ANOVA testing revealed a statistically significant interaction. Sample size was not calculated based on an a-priori power analysis.

## Results

### PAB increased right ventricular systolic pressure and induced right ventricular hypertrophy

**Fig 1:**
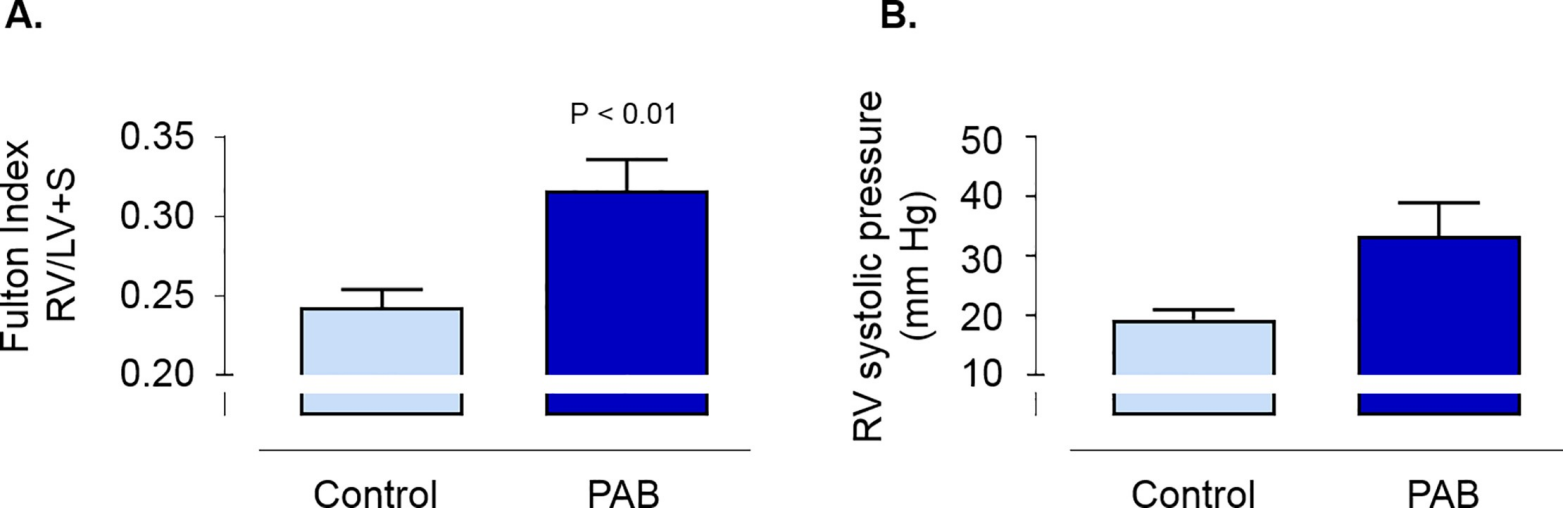
**The Fulton Index (A; weight of the right ventricle (RV) / weight of the left ventricle (LV) + septum) and RV systolic pressure (B) in mice after pulmonary artery banding (PAB) or sham surgery (control).** Data reported as mean ± SEM. Statistical differences determined by two-tailed unpaired Student’s t-test.

PAB resulted in right ventricular (RV) hypertrophy based on a 30% increase in the Fulton Index (Fig 1A). Hemodynamic assessment of RV systolic pressure demonstrated a non-statistically significant increase in the RV systolic pressure in the PAB mice compared to controls (Fig 1B, 33.3 ± 5.6 vs. 19.3 ± 1.7 mm Hg, p = 0.056). Body weight was measured at three time points across the study and there was no evidence that the PAB intervention adversely impacted weight gain, consistent with the experimental group not exhibiting any significant co-morbidities (Table 1).

**Table 1:** Weight trends in mice after pulmonary artery banding (PAB) or sham surgery (control).

	Control Mice (n = 7)	PAB Mice (n = 10)	p value
Weight (g)			
Time of PAB/sham surgery	25.1 ± 0.8	26.3 ± 0.8	0.31
Time of sleep study	25.9 ± 0.8	26.8 ± 1.2	0.54
Time of sacrifice	28.1 ± 0.6	27.8 ± 0.6	0.73

Data shown as mean ± SEM. Statistical differences determined by two-tailed unpaired Student’s t-test

### The PAB group had significantly increased NREM sleep time over the 24 hour period compared to control mice

**Fig 2:**
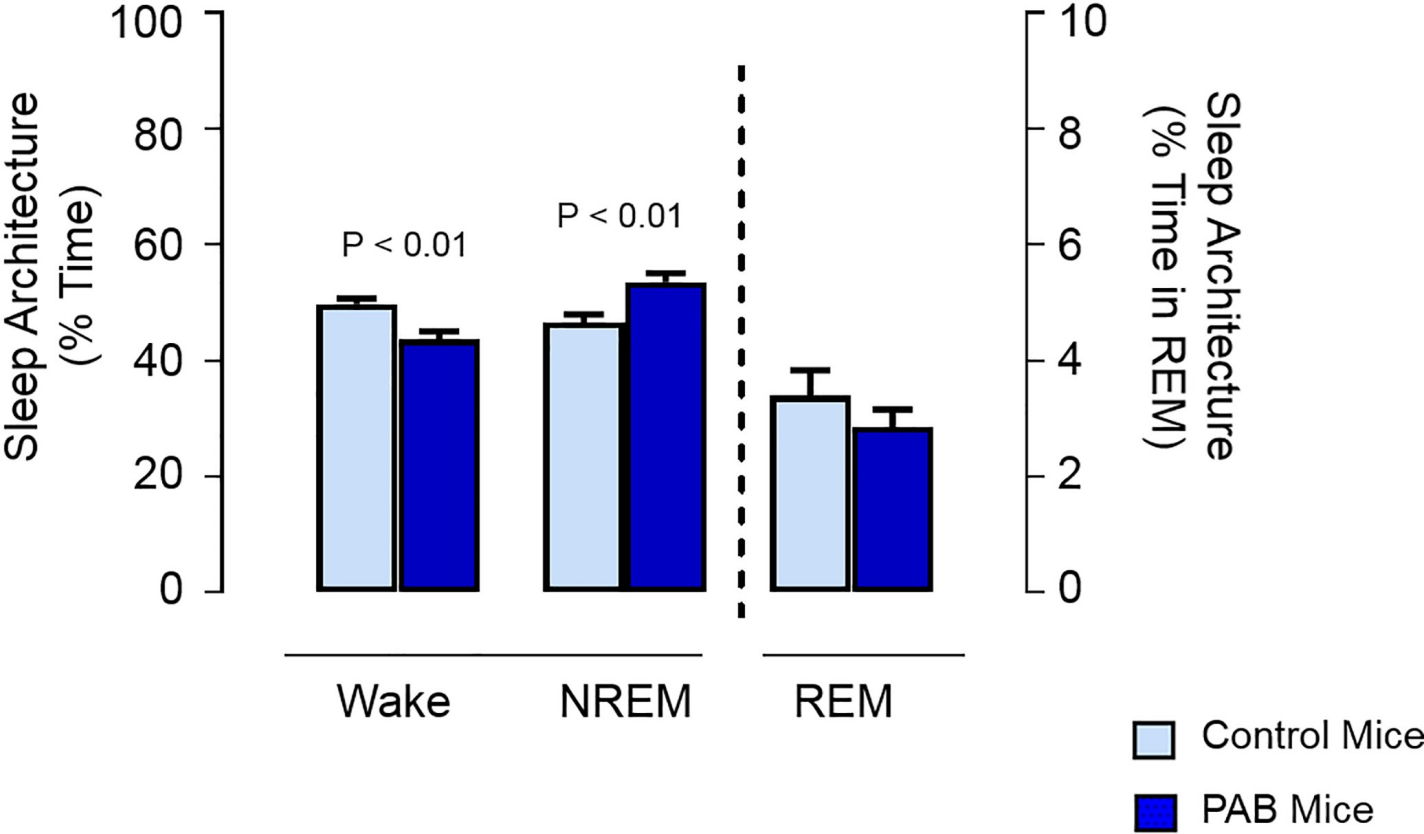
The 24-hour means ± SEM for percent time in Wake, NREM sleep, and REM sleep in mice after pulmonary artery banding (PAB) or sham surgery (control). The y-axis on the left refers to percentage of time for Wake and NREM sleep and the y-axis on the right refers to percentage of time for REM sleep. Statistical differences determined by two-tailed unpaired Student’s t-test.

The PAB group spent significantly more time in NREM sleep compared to the control group over a 24 hour period (Fig 2, 53.5 ± 1.5% vs. 46.6 ± 1.4%; p < 0.01). The increased NREM sleep time was noted in the setting of a significant decrease in Awake time for the PAB group and a similar overall percentage of time in REM sleep between the two groups.

### PAB increased NREM sleep time, impaired ability to cycle into REM sleep, and shortened time to resume sleep in the light/sleep period

**Fig 3:**
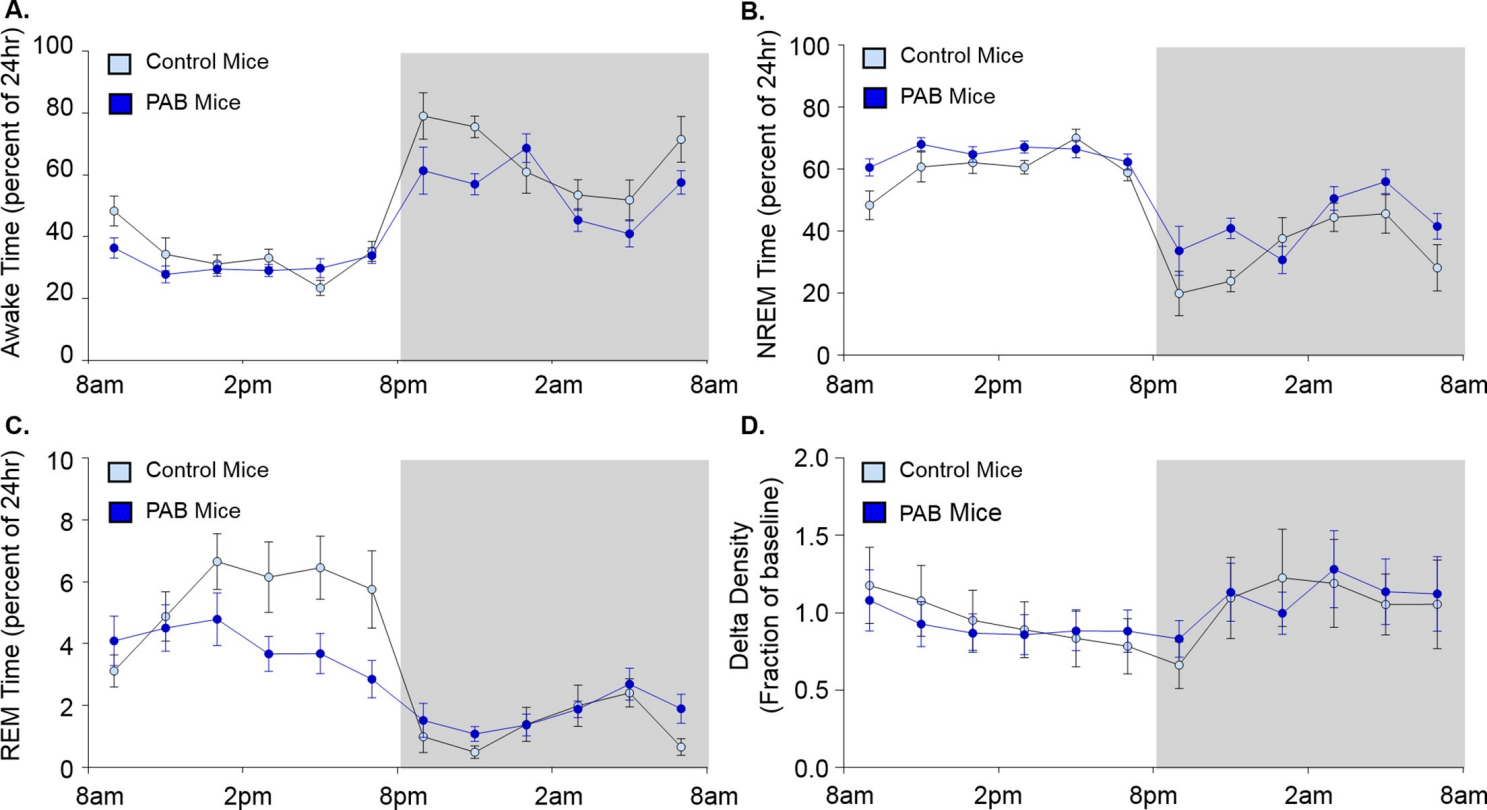
**Circadian patterns for Wake (A), NREM sleep (B), REM sleep (C), and Delta Density (D) in mice after pulmonary artery banding (PAB) or sham surgery (control).** Data reported as the mean ± SEM on a 2 hourly basis throughout the circadian period. The light region of each graph depicts the light period and the dark region of each graph depicts the dark period. Sleep stage is reported based on a percentage of time at each time-point. Delta density was averaged in two-hour increments and reported as a fraction of the 24-hour average in each mouse. Statistical differences determined by two-way ANOVA are reported in the text.

PAB mice exhibited increased NREM sleep time compared to the control mice during the light/sleep period (64.8 ± 1.2%/12hr vs. 60.6 ± 2.0%/12hr; Fig 3B; F(1, 15) = 5.1; p < 0.05). Despite increased NREM sleep time, PAB mice showed significant deficits in REM sleep time during the second half of the 12-hour light/sleep period (Fig 3C; p < 0.05 at 3, 5, and 7 pm and F(3.1, 46.5) = 3.6 for interaction term adjusted for sphericity; p < 0.05). The deficit in REM sleep time in the PAB mice was due to a decrease in the number of REM sleep bouts, rather than a decrease in the duration of REM sleep bouts (Table 2). Although arousals during the light/sleep period were not significantly different between groups, the time to resume sleep following an arousal was significantly reduced in PAB mice compared to control mice (Table 2). As expected, there was an overall reduction in delta density with time across the light/sleep period (Fig 3D; F(2.5, 38.4) = 20.5 for time adjusted for sphericity; p < 0.001), and a significant interaction between groups (Fig 3D; F(2.5, 38.4) = 4.2 adjusted for sphericity; p < 0.05) indicated that PAB mice were less able to dissipate delta density across the light/sleep period compared to control mice.

**Table 2:** Sleep architecture in mice after pulmonary artery banding (PAB) or sham surgery (control).

	Control Mice	PAB Mice
**Light period**		
Number of NREM Sleep Bouts	69.6 ± 6.3	61.1 ± 1.5
Duration of NREM Sleep Bouts (min)	6.2 ± 0.5	7.3 ± 0.4
Number of REM Sleep Bouts	37.0 ± 2.6	27.2 ± 3.3*
Duration of REM Sleep Bouts (min)	0.9 ± 0.1	0.8 ± 0.1
Number of arousals per hour of sleep	17.9 ± 0.4	16.1 ± 2.0
Time to Resume Sleep After an Arousal (min)	3.2 ± 0.4	2.3 ± 0.2*
**Dark period**		
Number of NREM Sleep Bouts	46.2 ± 5.8	46.4 ± 3.8
Duration of NREM Sleep Bouts (min)	4.2 ± 0.2	5.8 ± 0.5**
Number of REM Sleep Bouts	9.8 ± 1.6	13.4 ± 1.5
Duration of REM Sleep Bouts (min)	0.4 ± 0.1	0.5 ± 0.1
Number of arousals per hour of sleep	29.2 ± 3.5	25.0 ± 2.5
Time to Resume Sleep After an Arousal (min)	6.0 ± 1.2	6.6 ± 1.2

Data shown as mean ± SEM. NREM = Non-rapid eye movement sleep; REM = Rapid eye movement sleep. Statistical differences determined by two-tailed unpaired Student’s t-test on categorical variables and two-way ANOVA on continuous variables.

* p<0.05

** p<0.02

### PAB increased NREM sleep bout duration in the dark/active period

PAB mice exhibited a non-statistically significant increase in NREM sleep during the dark/active period compared to control mice (Fig 3B; 42.4 ± 3.1%/12hr vs. 33.3 ± 2.3%/12hr; F(1, 15) = 4.4; p = 0.053), associated with longer NREM sleep bouts compared to control mice (Table 2; p < 0.02) but no difference in the number of NREM sleep bouts. Despite a reduction in REM sleep during the light/sleep period, there was no evidence of REM sleep rebound during the dark/active period in PAB mice (Fig 3C). Delta density, arousals per hour of sleep, and time to resume sleep after arousal were not different between PAB and control mice (Fig 3D and Table 2) in the dark/active period.

## Discussion

Our study is the first to test in an animal model whether RV dysfunction can impair sleep architecture independent of obesity, SDB, and on the basis of a normal weight gain trajectory or other significant co-morbidities. Our data suggest a unique and internally consistent sleep phenotype in mice, which exhibit features of RV dysfunction in response to PAB. During the light/sleep period, RV dysfunction caused an increase in total NREM sleep time that was associated with a shorter time to resume sleep following an arousal and a decreased ability to cycle into REM sleep. When the above findings are placed in context of an attenuated dissipation of delta density, we conclude that chronic RV dysfunction impedes the transition from NREM to REM sleep, resulting in a ‘sleepy’ phenotype.

Evidence linking heart failure to increased sleepiness has been inconsistent in human studies, particularly in the context of objective and subjective parameters of sleepiness. Objective parameters measuring sleepiness and sleep architecture have demonstrated that patients with LV heart failure are sleepier than control populations [14, 23, 24], despite no apparent difference in subjective measures of sleepiness as determined by the Epworth Sleepiness Scale (ESS). Similarly, Javaheri et al. [25] and Kaneko et al. [26] report a lack of subjective sleepiness in patients with heart failure. In contrast, Artz et al. [15] showed that patients with a reduced LV ejection fraction have less subjective sleepiness, less sleep time, and a lower sleep efficiency compared to subjects without heart failure, and that these findings were independent of comorbid SDB. More recently, Mehra and colleagues [13] examined sleep in 26 subjects with stable LV heart failure and SDB. The mean sleep onset latency was significantly shorter in the heart failure group with SDB, despite no significant difference in subjective sleepiness; again highlighting a pattern of objective, but not subjective, sleepiness in patients with heart failure [13]. To our knowledge, no clinical studies have linked specific RV failure to disrupted sleep. However, patients with pulmonary hypertension exhibit a phenotype of increased nocturnal rest, as assessed by actigraphy [27], consistent with our findings of increased NREM sleep time in PAB mice during the light/sleep period. The difficulty in examining RV heart failure in isolation from other co-morbidities, and the disparity between objective versus subjective sleep outcomes, has limited our insight from clinical studies of heart failure and sleep.

Thus, our motivation was to use an animal model to determine the potential for a causal link between right heart failure and impairments in sleep architecture. We adopted a PAB intervention that induced elevated PA pressure and produced a RV-specific hypertrophy. An alternative intervention using aortic banding would have impacted both LV and RV function, potentially resulting in a more complex sleep phenotype. However, having now established interesting and internally consistent impairments in sleep architecture with PAB banding, there is now a framework for interpreting sleep responses in a model of aortic banding.

Although underlying mechanisms that link heart failure to impaired sleep architecture and sleepiness are unknown, pro-inflammatory responses may play a role through at least two pathways. First, in the form of a systemic inflammatory response of cardiac origin. Mehra et al. [13] reported a significant positive correlation between cytokine levels (TNF-alpha and hs-IL-6) and objective measures of sleepiness, consistent with pre-clinical studies showing TNF-alpha increases non-REM sleep time [28, 29] and that inhibition of TNF-alpha and other pro-inflammatory markers can reduce deep sleep time [30, 31]. Specifically with relevance to the pre-clinical model used in our study, circulating levels of TNF-alpha are elevated in patients with advanced right-sided heart failure and correlate with increasing severity of peripheral edema [32]. A second possible pro-inflammatory mechanisms is that activation of sympathetic afferents in the remodeling myocardium can impact cytokine and inflammatory responses in the CNS. Indeed, Francis et al. [33] have shown that experimental myocardial infarction in rats can increase pro-inflammatory cytokines in the hypothalamus, an important CNS structure involved in sleep-wake homeostasis. Lastly, the reduction in REM sleep during the latter half of the light/sleeping period (together with the increase in NREM sleep) may be related to increased cortisol levels in the PAB mice as described by Friess and colleagues in human studies [34]. Further studies investigating pro-inflammatory responses, or other potential mechanisms, would be a productive corollary to our demonstration of a causal relationship between RV dysfunction and a phenotype of impaired sleep architecture.

## Limitations

There are several potential limitations in our study design. We did not perform an assessment of inflammatory cytokines in this exploratory study. Lack of such data limits interpretability of causal mechanisms as suggested above. We also did not measure the impact that pulmonary artery banding had on the respiratory mechanics, control of breathing, or systemic hemodynamics in our mouse model. There are a number of unanticipated hemodynamic changes from pulmonary artery banding which may have had impact on the results. We did not perform hemodynamic assessments during the sleep studies nor did we assess for sleep-disordered breathing to account for the potential role such differences may have in the sleep architecture between the pulmonary artery banded and control mice. Additionally, the time delay between sleep architecture assessment and sacrifice for hemodynamic assessment may impact data obtained for RV dysfunction between the groups.

## Clinical implications and conclusions

Sleep abnormalities and congestive heart failure are strongly linked and confounded by a number of variables. In our mouse model of RV failure, we establish a causal relationship between isolated RV failure and increased non-REM sleep time, decreased ability to cycle into REM sleep, and reduced time to resume sleep during the light/sleeping period, consistent with a phenotype of increased ‘sleepiness.’ Our results highlight that even in the absence of SDB, or other common clinical co-morbidities, RV failure impacts sleep architecture and daytime sleepiness. Taking our data together with the available human studies, we propose that objective, rather than subjective, measures of sleepiness may better inform clinical decisions regarding therapeutic interventions to normalize sleep in patients with heart failure, to improve quality of life, and to protect against the development of sleep-dependent co-morbidities such as weight gain, pro-inflammatory states, and metabolic disturbances.

## Supporting information

S1 Fig Data for Fig 1. (XLSX)

S2 Fig Data for Fig 2. (XLSX)

S3 Fig Data for Fig 3. (XLSX)

S1 Table Data for Table 1. (XLSX)

S2 Table Data for Table 2. (XLSX)
