# Correction: A phenotype of increased sleepiness in a mouse model of pulmonary hypertension and right ventricular hypertrophy

**DOI:** 10.1371/journal.pone.0218401

**Published:** 2019-06-10

**Authors:** Eric M. Davis, Jeffrey J. Baust, Brett J. O’Donnell, Faraaz A. Shah, Angela McDowell, Lanping Guo, Christopher P. O’Donnell

In the Funding section, the grant number from the funder National Institutes of Health to EMD is listed incorrectly. The correct grant number is: T32 HL082610.

## References

[1] Davis EM, Baust JJ, O’Donnell BJ, Shah FA, McDowell A, Guo L, et al (2018) A phenotype of increased sleepiness in a mouse model of pulmonary hypertension and right ventricular hypertrophy. PLoS ONE 13(12): e0208540 10.1371/journal.pone.0208540 30532231 PMC6286175

